# Older Adults’ Exposure to Ageism in Daily Life – On the Role of TV, Social Media, and Neighborhood Composition

**DOI:** 10.1093/geroni/igaf122.1845

**Published:** 2025-12-31

**Authors:** Juhyeong Lee, Yoonseok Choi, Christiane Hoppmann

**Affiliations:** The University of British Columbia, Vancouver, British Columbia, Canada; Simon Fraser University, Vancouver, British Columbia, Canada; UBC, Vancouver, British Columbia, Canada

## Abstract

Experiences of ageism undermine well-being in old age but the underlying everyday mechanisms are not well understood. Our study focused on three potential exposure contexts: a) TV, b) social media, and c) neighborhood composition that may contribute to the experience of everyday ageism. We aimed to investigate the association between TV viewing, social media use, and neighborhood age composition with ageism reports. We analyzed up to 14 days of end of day diaries from 76 older adults living in Canada (Age: M = 72.01, SD = 9.47; 80% female), with data collection still ongoing. Participants reported their daily TV viewing, social media use, and exposure to ageism each evening over fourteen consecutive days. To determine the age composition of the neighborhoods where participants resided, we used the first three digits of their postal codes. We pre-registered the study on the Open Science Framework. We employed hierarchical linear models to account for the nested data structure. As hypothesized, result indicate that greater overall social media use was significantly associated with greater experiences of ageism in daily life (b = 0.141, p < .01). However, TV viewing and neighborhood age composition showed no significant association with ageism over and above social media use. The results suggest that social media serves as a primary context where older adults experience ageism in their daily lives. Future analyses will replicate the findings using the complete dataset and a larger sample, and will explore potential moderators of the social media–everyday ageism nexus.

